# Opt‐in or opt‐out health‐care communication? A cross‐sectional study

**DOI:** 10.1111/hex.13198

**Published:** 2021-03-24

**Authors:** Vivien Tong, Ines Krass, Stephen Robson, Parisa Aslani

**Affiliations:** ^1^ The University of Sydney School of Pharmacy Faculty of Medicine and Health Pharmacy and Bank Building (A15) The University of Sydney Camperdown NSW Australia; ^2^ Healthy Thinking Group St Leonards NSW Australia; ^3^ Present address: Youmeds Belrose NSW Australia

**Keywords:** attitude to health, consumer health information, health communication, health literacy, patient education as topic

## Abstract

**Background:**

Patients need medication and medical condition‐related information to better self‐manage their health. Health‐care professionals (HCPs) should be able to actively provide information outside of one‐on‐one consultations; however, patient consent may be required.

**Objective:**

To investigate the Australian public's preferences, and factors that may influence their preferences, towards an opt‐in versus an opt‐out approach to health communication.

**Design:**

A cross‐sectional study using a structured questionnaire administered via Computer‐Assisted Telephone Interviewing.

**Setting and participants:**

Participants across Australia who were adults, English‐speaking and had a long‐term medical condition.

**Main outcome measures:**

Preferences for opt‐in vs opt‐out approach to receiving follow‐up tailored information.

**Results:**

A total of 8683 calls were made to achieve the required sample size of 589 completed surveys. Many (346/589; 58.7%) indicated that they were interested in receiving tailored, ongoing follow‐up information from their HCP. Nearly half (n = 281; 47.7%) preferred an opt‐in service and 293/589 (49.7%) an opt‐out service for receiving follow‐up information. Reasons for preferring an opt‐in service were being in control of the information received (n = 254); able to make a decision that is best for them (n = 245); opt‐in service would save time for HCPs (n = 217); they may not want or need the information (n = 240). Many (n = 255) felt that an opt‐out service should be part of the normal duty of care of their HCP and believed (n = 267) that this approach would ensure that everyone has access to information.

**Conclusions:**

Respondents were interested in receiving tailored information outside of consultation times. However, preferences for an opt‐in or opt‐out approach were divided.

## 1 INTRODUCTION

Information relating to medical conditions and medicines is important to help enable patients to better self‐manage their health.^1^ Health and medicines information may be accessed by patients in a number of ways including, but not limited to, spoken information delivered by health‐care professionals (HCPs) either during a consultation, or over the phone; through online platforms such as websites, apps and social networking sites; and written forms such as patient information leaflets, newsletters and booklets. However, since patients’ preferences and needs for information are diverse and vary throughout their patient journey, a ‘one‐size‐fits‐all’ approach is unlikely to cater for everyone.^2^

Patients desire tailored information^2^, ^3^ and are entitled to receive relevant health and medicines information that is appropriate for their needs.^4^ It is therefore incumbent upon HCPs to identify and deliver such targeted health information. However, research has shown that patients’ information needs have not always been well met at different points along the health‐care continuum.^5^ HCPs typically communicate health and medicines information at face‐to‐face consultations. However, consultation times can vary^6^ which may then impact the degree to which complete, necessary information is received by patients in a timely manner. Furthermore, information that is provided during consultations may not be remembered,^7^ or easily understood and enacted.^8^, ^9^ Although more patients are seeking information themselves via the Internet and social networking sites, the quality, accuracy and relevance of the information sourced can vary.^10^ Furthermore, information‐seeking behaviour may differ between patients, where certain factors such as medical condition, health literacy level and health locus of control can influence the likelihood of patients reading and/or sourcing information, such as written medicines information.^11^

One approach to ensuring that patients receive appropriate, relevant and quality information, tailored to their individual needs throughout their patient journey, is for HCPs to actively provide information, not only during face‐to‐face consultations, but regularly using multi‐modal communication channels as part of patient follow‐up.^12^ However, patient consent may be needed for provision of ongoing health information by HCPs outside of consultation times. For example, in Australia, according to the Privacy Act 1988**—**Australian Privacy Principles, patients must provide consent for a registered HCP to use their personal information to provide them with proactive support and advice on an ongoing basis^13^; however, depending on the context and a case‐by‐case basis, implied consent may be acceptable for certain communications.^14^ Seeking consent upfront is regarded as an opt‐in approach and may limit the type and nature of support and information received by patients. However, in countries such as the United States, where prior consent from patients is not required, HCPs may send additional information related to treatment/health care.^15^ Patients not wishing to receive such information would need to ‘opt‐out’ based on the Standards for Privacy of Individually Identifiable Health Information.^16^ Health‐care systems with an ‘opt‐out’ approach may therefore differ in how they facilitate the provision of tailored and timely information by HCPs outside consultation times.

Both opt‐in and opt‐out approaches have their merits, and it is important to consider patients’ needs as well as preferences in receiving information when determining an approach for a country's health‐care system. In exploring patient preferences for follow‐up information, previous research has found great diversity, suggesting that a tailored approach may lead to increased patient satisfaction.^17^ For example, the findings of a qualitative study conducted with a sample of Australian patients showed that while participants were receptive to receiving tailored information about their medicines and/or medical conditions from their HCP on an ongoing basis, there was variation in expressed preferences for the method of information delivery, frequency and type of content.^18^ The concept of timely and tailored information provision that meets patients’ needs on an ongoing basis underpins the rationale for this research. This study aimed to investigate the Australian public's preferences and factors that may influence their preferences towards an opt‐in versus an opt‐out approach to the provision of information by HCPs on an ongoing basis.

## 2 METHODS

Ethics approval for the conduct of this study was granted by the institution's Human Research Ethics Committee (project number 2017/164; approval date 21 April 2017).

A cross‐sectional study was conducted using a structured questionnaire administered via Computer‐Assisted Telephone Interviewing (CATI). CATI was the preferred method for survey administration as it is considered more efficient in comparison to paper‐based questionnaires. Data are entered in real time,^19^ thus facilitating greater precision in survey administration and data management, reductions in the incidence of incomplete responses/missing values and timely output of data deliverables supported by quality assurance processes.^19^

### 2.1 Questionnaire development

CATI questionnaire development was informed by a previous qualitative study.^18^ The previous study consisted of a series of 6 focus groups (n = 46 participants in total) conducted in metropolitan Sydney, Australia, with adult, English‐speaking participants who had at least one long‐term medical condition. A semi‐structured focus group protocol addressed discussion topics relating to participants’ health and/or medicine information sources; and their perspectives on receiving information from their HCP(s), and systems and consenting process to facilitate receiving such information. Participants emphasized the importance of tailored information and held mixed views on how consent should be obtained for HCP‐initiated information provision.

Based on the themes and subthemes derived from the thematic analysis of the verbatim focus group transcripts, the items in the CATI structured questionnaire covered:

- Previous experiences, opinions and preferences about receiving follow‐up tailored information about medical conditions and/or treatments from HCPs outside of consultation times on a regular basis;
- Patient perspectives regarding whether the above should be an opt‐in or opt‐out process;
- Patient preferences for information in response to scenarios representing different time points in the patient medication‐taking continuum; and
- Demographics.

The structured questionnaire was piloted to establish its clarity, face and content validity, and feasibility of administration. Two HCPs (a doctor and a pharmacist/researcher) who were external to the research team initially pilot‐tested the questionnaire. Subsequent pilot tests were conducted with 4 non‐HCPs/consumers. All pilot tests were conducted sequentially by telephone to simulate conditions under which the questionnaire would be administered (CATI). Piloting and subsequent revisions were conducted iteratively until no further improvements to the questionnaire were deemed necessary by the research team and the questionnaire could be administered in less than 15 minutes in consideration of the impact of survey length on respondent fatigue and response rates.^20^ The final survey consisted of 4 sections (Table 1).

**Table 2:**
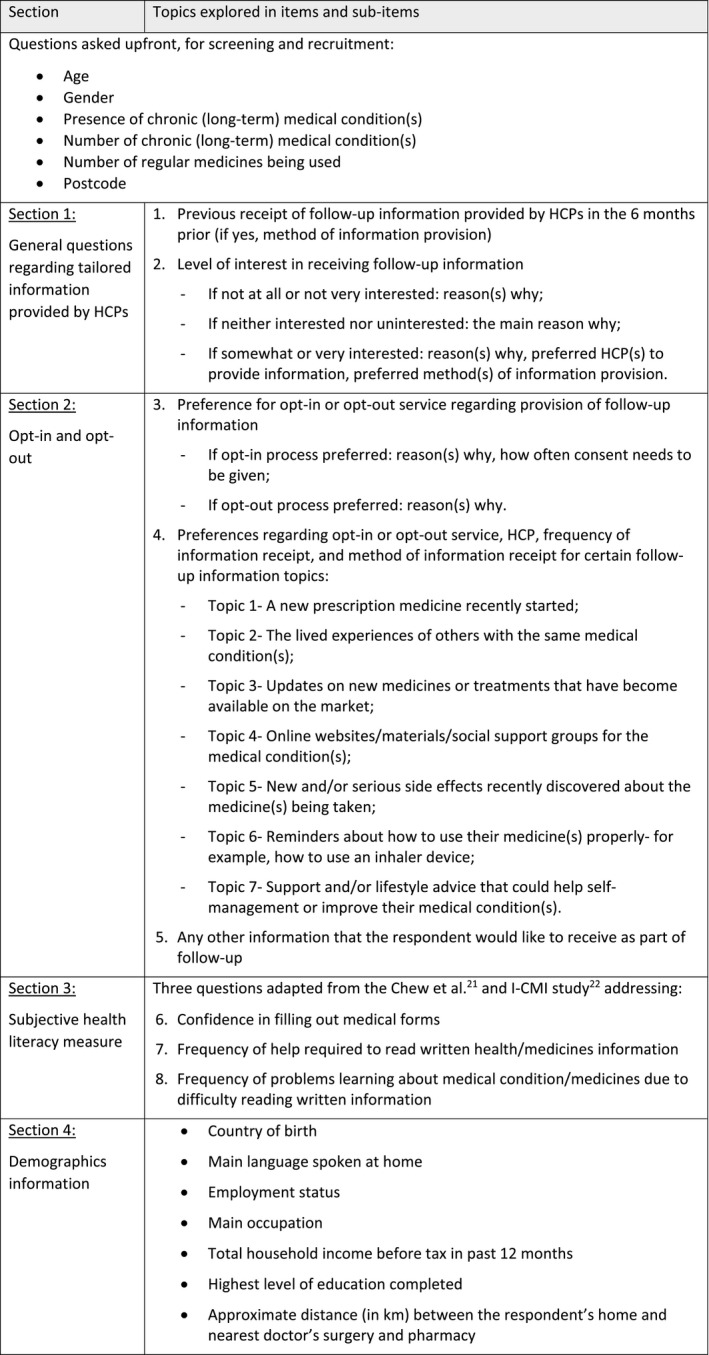
Summary of survey sections

### 2.2 Sample size and sample stratification

The sampling strategy involved several steps. The initial step involved the calculation of the sample size required to measure an estimated prevalence of the preference for receiving opt‐out health information among members of the Australian public, estimated as 50% from a previous study.^18^ For a simple random sample^23^ with a confidence level of 95%, population percentage as 50%, and degree of precision of 5%, the minimum sample size required was 385 participants. This was inflated to 500 to account for sample stratification to ensure a nationally representative sample with adequate representation from subpopulations.

The stratification strategy used to generate interlocked quotas was based on data from the Australian Bureau of Statistics (ABS). The four key parameters taken into consideration when calculating the sample quotas required for stratification were:

1. State/Territory population sizes (to obtain a nationally representative sample of the Australian population)^24^;
2. Remoteness Areas (population proportions residing in Major Cities, Regional (Inner and Outer), and Remote (Remote and Very Remote) areas in Australia)^25^;
3. Index of Relative Socio‐economic Disadvantage (IRSD) (sampling was intended to encompass the full spectrum of the IRSD; IRSD deciles linked to Postal Areas were stratified into low, middle, and high IRSD groups for the sampling strategy)^26^; and
4. Age (in light of the prevalence of long‐term medical conditions in the Australian population by age).^27^

In addition, an equal distribution of male and female respondents was also included as part of the overall target quota, as per ABS data. However, due to the study inclusion criteria and CATI survey methods utilized, a skew towards a larger number of respondents aged 55 years and older was anticipated.

As the generated sample yielded a low proportion of the population residing in Remote Australia (n = 11 of a total of 500), oversampling of respondents within this group was undertaken to ensure an adequate sample size to detect a difference in preferences for an opt‐in or opt‐out approach between groups of participants by Remoteness Area (Major Cities, Regional Australia, and Remote Australia). Based on the calculations to determine the required sample size sufficient to detect a difference in proportions,^28^ with a confidence level of 95%, 80% power, and the sample proportions for each group estimated to be 50% and 70%, respectively, the minimum sample size required would be 91. Thus, in order to meet this minimum, the required number of respondents residing in Remote Australia (inclusive of Remote and Very Remote) was oversampled and increased to n = 100. Thus, this constituted the need for a further 89 respondents to be recruited in addition to the initial proposed sample size of 500 respondents.

The final sample size, determined *a priori*, was 589 respondents, with all sampling quotas adjusted where necessary to reflect this sample size. It should be noted that there was some interlocking of these quotas, which was accounted for in the recruitment process to ensure that all stratification quotas would be met once all respondents were recruited.

### 2.3 Nationwide administration of the survey

The CATI survey was administered nationally across all major Australian States and Territories, between May and August 2017. Participation in the study was completely voluntary and respondents received no financial reimbursements.

Individuals were eligible to participate in the study if they were adults (18 years and above); English‐speaking (ie did not require the assistance of an interpreter to participate); and had a long‐term medical condition, that is an ongoing medical condition that had been experienced for at least 6 months, which required treatment with at least one medicine.

A market research company, contracted by the research team, contacted potential participants by telephone. Phone numbers were generated using a predictive dialler system via a random digit dialling process, which automatically removed wrong numbers (eg. numbers with no pulse tone) and only delivered live phone numbers to the interviewers. Postcodes linked to these generated phone numbers were used to help ensure nationwide administration and that stratified sample quotas were met. Once consent had been obtained and eligibility verified, the survey was administered by the interviewer and participant responses were recorded.

### 2.4 Data analysis

Survey responses were analyzed using IBM^®^ SPSS^®^ Statistics (Version 24). Initially, the data file was examined, and variables were recoded where necessary in preparation for further analysis.

Due to the CATI process, there were no missing data as a relevant response had to be recorded by the interviewer in relation to each question during the survey administration process. In the case of a participant refusing to provide a response, the response was classified as a missing value.

Descriptive statistics were compiled to examine the demographics and other questionnaire items. The Mann‐Whitney *U* test was used to compare continuous variables (which were not normally distributed) between two groups (eg. those who favored an ‘opt‐in’ approach versus those who favored an ‘opt‐out’ approach). Chi‐squared tests were conducted for categorical variables of interest. McNemar's test was used to compare proportions for paired categorical data, for example broad opt‐in/opt‐out preferences and subsequent preferences for each individual information topic. The significance level in the statistical analyses was set *a priori* at *P* = 0.05.

## 3 RESULTS

A total of 8683 telephone calls were made to achieve the required sample size of 589 individuals who completed the survey, yielding an overall response rate of 6.8%. However, the survey completion rate, calculated as the proportion of respondents who completed the survey out of the total number of eligible respondents who commenced the survey (n = 654), was 90.1%. A total of 6519 people refused to participate by hanging up without listening to the introduction (n = 2859), or after the introduction (n = 3595), or during the survey (n = 65); and 1449 participants did not meet the inclusion criteria. Overall, the original sample stratification target quotas were met, with the exception of the target age quotas and broad gender quotas (Table 2).

**Table 2:** Sample stratification and relevant overall sampling quotas achieved

Sampling stratification factor		Target quota	Frequency (%) (n = 589)
State / Territory	Australian Capital Territory	8	8 (1.4)
	New South Wales	167	167 (28.4)
	Northern Territory	22	22 (3.7)
	Queensland	124	124 (21.1)
	South Australia	46	46 (7.8)
	Tasmania	13	13 (2.2)
	Victoria	127	127 (21.6)
	Western Australia	82	82 (13.9)
Remoteness Area	Major cities	356	356 (60.4)
	Regional	133	133 (22.6)
	Remote	100	100 (17.0)
Index of Relative Socio‐economic Disadvantage	Low (Deciles 1‐4)	220	220 (37.4)
	Middle (Deciles 5‐7)	178	178 (30.2)
	High (Deciles 8‐10)	191	191 (32.4)
Age	18‐34 years	120	36 (6.1)
	35‐54 years	174	119 (20.2)
	55 years and above	295	434 (73.7)
Gender	Male	294	258 (43.8)
	Female	295	331 (56.2)

### 3.1 Respondent demographics and subjective health literacy

Respondent demographics are shown in Table 3. The median number of ongoing medical conditions that respondents had that had lasted for more than 6 months and required treatment with medicine(s) was 2 (n = 588, IQR = 1‐3), with a median of 4 medicines used regularly (n = 589, IQR = 2‐6). The median approximate distance (in kilometers) between the respondent's home and the nearest doctor's surgery and pharmacy was 2.0 km (n = 586, IQR = 1.0‐5.0) and 1.5 km (n = 587, IQR = 1.0‐3.0), respectively.

**Table 3:** Summary of respondent demographics (n = 589)

Demographic factor		Frequency (%)
Country of birth	Australia	452 (76.7)
	Other	136 (23.1)
	Refused to answer	1 (0.2)
Main language spoken at home	English	575 (97.6)
	Other	14 (2.4)
Employment status	Working full‐time	124 (21.1)
	Working part‐time	98 (16.6)
	Retired or unemployed	344 (58.4)
	Carer / home duties	12 (2.0)
	Student / volunteer	10 (1.7)
	Refused to answer	1 (0.2)
Total household income before tax or any other deductions over the past 12 months (in Australian dollars)	< $25,000	132 (22.4)
	Between $25,000 and $50,000	139 (23.6)
	Between $50,000 and $100,000	114 (19.4)
	Between $100,000 and $150,000	58 (9.8)
	> $150,000	56 (9.5)
	Refused to answer	66 (11.2)
	Don't know	24 (4.1)
Highest level of education	Year 11 or below	193 (32.8)
	Year 12	69 (11.7)
	TAFE or college	133 (22.6)
	University degree or higher	192 (32.6)
	Refused to answer	2 (0.3)

A majority (87.6%) were extremely or quite confident with filling out forms on their own, based on their self‐reported subjective health literacy. Most reported never requiring help from someone with reading written health and/or medicine information (78.9%), as well as never experiencing problems learning about their medical condition or medicines due to difficulty with reading written information (83.9%).

### 3.2 Previous receipt of information outside of consultation times and level of interest in receiving follow‐up information

A total of 530 (90%) respondents indicated that they had not received follow‐up information from their HCPs in the 6 months preceding their study participation. However, the majority (346/589; 58.7%) indicated that they were either somewhat or very interested in receiving tailored, ongoing follow‐up information from their HCP. Of those who expressed interest, the most common reason was that they wanted their HCP to adopt a more proactive approach with respect to information provision. Interestingly, only one in three respondents reported having received insufficient information from their HCP, as a reason for their interest in follow‐up information.

Of those interested in receiving follow‐up, the majority expressed a preference for their GP to provide this information, with about half preferring pharmacists. Telephone (155/346; 44.8%) and email (188/346; 54.3%) were the predominant preferred methods for receiving follow‐up information from their preferred HCP(s).

A lack of interest in receiving such information was expressed by 34.3% of respondents (not at all or not very interested). The following reasons were provided: they received all necessary information during the consultation, they were currently adherent to their prescribed medicine(s), and/or they asked their HCP if they wanted information. Those who reported needing help to read written health and/or medicine information (*P* < 0.001) and those who reported having had problems learning about their medical condition or medicines due to difficulty reading written information (*P* < 0.001) were more likely to be interested in receiving follow‐up information.

### 3.3 Preference for an opt‐in or opt‐out approach to receiving ongoing follow‐up information

When asked if an opt‐in or opt‐out service would be preferred for the provision of ongoing follow‐up information outside of consultation times, 281/589 respondents (47.7%) indicated that they would prefer an opt‐in service and 293/589 respondents (49.7%) preferred an opt‐out service. Fifteen respondents refused to provide a response (2.5%).

### 3.4 Opt‐in

The majority who preferred an opt‐in service indicated that all the proposed reasons for preferring an opt‐in service listed in the questionnaire applied to them. Respondents wished to be in control of the information they received (n = 254; 90.4%); they felt that they were able to make a decision that is best for them (n = 245; 87.2%); the opt‐in service would help save time for HCPs (n = 217; 77.2%); and they may not want or need the information being provided (n = 240; 85.4%).

There was no clear trend as to the preferred frequency of consent that would need to be given for the provision of ongoing follow‐up information. A range of responses were provided, such as once‐off only (n = 72; 25.6%); every time they see their HCP (n = 65; 23.1%); every 6 months (n = 62; 22.1%); or every 12 months (n = 57; 20.3%).

### 3.5 Opt‐out

Of those who preferred ongoing follow‐up information provision to be an opt‐out service (n = 293), the majority agreed that all the proposed reasons for preferring an opt‐out service applied to them. A total of 255 (87.0%) stated that an opt‐out service should be part of the normal duty of care of their HCP; 210 (71.7%) reported that they already sign forms when seeing their HCP, so this should automatically be a part of that; 267 (91.1%) believed that this approach would ensure that everyone gets access to information on an ongoing basis; and 275 (93.9%) felt that by experiencing it first, they can make an informed choice about whether it is right for them.

### 3.6 Associations between demographic variables and broad opt‐in/opt‐out preferences for receiving follow‐up information

Those who were interested in receiving information were more likely to prefer an opt‐out approach for the receipt of follow‐up information (*P* = 0.001) (Table 4). No other associations were found.

**Table 4:** Univariate statistics relating to preferences for broad opt‐in or opt‐out provision of follow‐up information

Variable		Opt‐in		Opt‐out		*P*‐value
		n	%	n	%	
Previous receipt of information outside of consultation times from HCP^a^ (n = 574)						
Yes		26	9.3	33	11.3	0.512
No		255	90.7	260	88.7	
Total		281		293		
Level of interest in receiving follow‐up information^b^ (n = 573)						
	N (%)					
Interested	342 (59.7)	136	48.6	206	70.3	**0.001**
Not interested / Neither interested nor uninterested	231 (40.3)	144	51.4	87	29.7	
N (%)		280 (48.9)		293 (51.1)		
Highest level of education^a^ (n = 572)						
Year 12 or below		119	42.7	135	46.1	0.460
TAFE / College / University degree / Higher		160	57.3	158	53.9	
Total		279		293		
Age^a^ (n = 574)						
18‐54 years		75	26.7	80	27.3	0.943
55 + years		206	73.3	213	72.7	
Total		281		293		
Gender^a^ (n = 574)						
Male		128	45.6	121	41.3	0.345
Female		153	54.4	172	58.7	
Total		281		293		
Remoteness Area^a^ (n = 574)						
Major cities		167	59.4	178	60.8	0.929
Regional		66	23.5	65	22.2	
Remote		48	17.1	50	17.1	
Total		281		293		
IRSD^a^ (n = 574)						
Low (Deciles 1‐4)		100	35.6	114	38.9	0.095
Middle (Deciles 5‐7)		96	34.2	76	25.9	
High (Deciles 8‐10)		85	30.2	103	35.2	
Total		281		293		
Country of birth^a^ (n = 573)						
Australia		206	73.6	235	80.2	0.074
Other		74	26.4	58	19.8	
Total		280		293		
Employment status^a^ (n = 573)						
Working full‐time		52	18.6	70	23.9	0.476
Working part‐time		48	17.1	49	16.7	
Retired or unemployed		169	60.4	164	56.0	
Carer / Home duties / Student / Volunteer		11	3.9	10	3.4	
Total		280		293		
Total household income in previous 12 months^a^ (n = 487)						
<$25,000		67	28.6	62	24.5	0.506
$25,000‐$150,000		139	59.4	163	64.4	
>$150,000		28	12.0	28	11.1	
Total		234		253		
Subjective health literacy^c^ (n = 574)						
Confident with forms		(Mdn = 5, IQR = 1)		(Mdn = 5, IQR = 1)		0.218
Help to read		(Mdn = 1, IQR = 0)		(Mdn = 1, IQR = 0)		0.959
Problems learning		(Mdn = 1, IQR = 0)		(Mdn = 1, IQR = 0)		0.120
Number of long‐term medical conditions^c^ (n = 573)						
		(Mdn = 2, IQR = 1)		(Mdn = 2, IQR = 2)		0.153
Number of regular medicines^c^ (n = 574)						
		(Mdn = 4, IQR = 4)		(Mdn = 4, IQR = 4)		0.220
Access to HCP‐ distance between respondent's home and nearest doctor's surgery^c^ (n = 571)						
		(Mdn = 2, IQR = 4)		(Mdn = 2, IQR = 4)		0.318
Access to HCP‐ distance between respondent's home and nearest pharmacy^c^ (n = 572)						
		(Mdn = 1, IQR = 2.2)		(Mdn = 1.5, IQR = 4)		**0.039**

^a^ Chi‐square test (with continuity correction *P*‐value reported for 2 x 2 contingency tables, where relevant).

^b^ McNemar's test.

^c^ Mann‐Whitney *U* test.

### 3.7 Preferences for receiving specific follow‐up information

Respondents were requested to indicate their preferences regarding 7 specific topics of information (Table 1) proposed to be provided as part of follow‐up (Table 5). Overall, for each of the information topics, the majority of respondents preferred their GP to be the provider of this information. The proportion who selected pharmacists as their preferred HCP to provide the topic‐specific follow‐up information was highest for reminders regarding medicine use (n = 194, 36.5%) in comparison to the other information topics.

**Table 5:** Summary of responses given in relation to the proposed provision of 7 specific information topics as part of follow‐up information

			Topic 1	Topic 2	Topic 3	Topic 4	Topic 5	Topic 6	Topic 7
			New prescription medicine	Lived experiences	Update on new treatments	Online information	New or serious side effects	Reminders on medicines use	Lifestyle advice
Variable	Responses		n (%)	n (%)	n (%)	n (%)	n (%)	n (%)	n (%)
Opt‐in versus opt‐out	Opt‐in service		228 (38.7)	296 (50.3)	244 (41.4)	315 (53.5)	150 (25.5)	274 (46.5)	278 (47.2)
	Opt‐out service		330 (56.0)	205 (34.8)	312 (53.0)	204 (34.6)	420 (71.3)	257 (43.6)	271 (46.0)
	Not interested in receiving^a^		29 (4.9)	84 (14.3)	33 (5.6)	69 (11.7)	18 (3.1)	58 (9.8)	40 (6.8)
	Refused to answer		2 (0.3)	4 (0.7)	0 (0)	1 (0.2)	1 (0.2)	0 (0)	0 (0)
	Total		589	589	589	589	589	589	589
Preferred HCP	GP (general practitioner)		393 (70.2)	380 (75.2)	431 (77.5)	366 (70.4)	424 (74.3)	270 (50.8)	411 (74.9)
	Pharmacist		117 (20.9)	59 (11.7)	74 (13.3)	58 (11.2)	95 (16.6)	194 (36.5)	43 (7.8)
	Nurse		13 (2.3)	23 (4.6)	9 (1.6)	44 (8.5)	8 (1.4)	41 (7.7)	55 (10.0)
	Other	Specialist	24 (4.3)	24 (4.8)	32 (5.8)	22 (4.2)	36 (6.3)	12 (2.3)	14 (2.6)
		Prescriber	8 (1.4)	3 (0.6)	6 (1.1)	5 (1.0)	5 (0.9)	7 (1.3)	3 (0.5)
		Support group / organization	0 (0)	6 (1.2)	1 (0.2)	11 (2.1)	0 (0)	1 (0.2)	5 (0.9)
		Other	4 (0.7)	10 (2.0)	3 (0.5)	13 (2.5)	3 (0.5)	6 (1.1)	17 (3.1)
	Refused		1 (0.2)	0 (0)	0 (0)	1 (0.2)	0 (0)	0 (0)	1 (0.2)
	Total		560	505	556	520	571	531	549
Frequency of receipt	Every week		25 (4.5)	11 (2.2)	17 (3.1)	13 (2.5)	79 (13.8)	18 (3.4)	23 (4.2)
	Every month		103 (18.4)	91 (18.0)	109 (19.6)	112 (21.5)	108 (18.9)	109 (20.5)	123 (22.4)
	Every 3 to 6 months		269 (48.0)	288 (57.0)	266 (47.8)	280 (53.8)	144 (25.2)	277 (52.2)	294 (53.6)
	Other	Every 12 months	27 (4.8)	41 (8.1)	32 (5.8)	46 (8.8)	9 (1.6)	48 (9.0)	46 (8.4)
		Ad hoc / when necessary or appropriate	81 (14.5)	38 (7.5)	48 (8.6)	35 (6.7)	53 (9.3)	42 (7.9)	34 (6.2)
		As soon as possible / once available	21 (3.8)	11 (2.2)	66 (11.9)	11 (2.1)	161 (28.2)	7 (1.3)	9 (1.6)
		Other	29 (5.2)	25 (5.0)	18 (3.2)	20 (3.8)	17 (3.0)	28 (5.3)	19 (3.5)
	Refused		5 (0.9)	0 (0)	0 (0)	3 (0.6)	0 (0)	2 (0.4)	1 (0.2)
	Total		560	505	556	520	571	531	549
Method of information provision	Telephone		131 (23.4)	115 (22.8)	124 (22.3)	96 (18.5)	199 (34.9)	132 (24.9)	116 (21.1)
	Email		277 (49.5)	256 (50.7)	284 (51.1)	298 (57.3)	235 (41.2)	258 (48.6)	299 (54.5)
	SMS		57 (10.2)	50 (9.9)	54 (9.7)	38 (7.3)	60 (10.5)	47 (8.9)	40 (7.3)
	Other	Mail / post	55 (9.8)	49 (9.7)	52 (9.4)	50 (9.6)	40 (7.0)	51 (9.6)	55 (10.0)
		Face‐to‐face	31 (5.5)	30 (5.9)	36 (6.5)	27 (5.2)	31 (5.4)	40 (7.5)	35 (6.4)
		Other	9 (1.6)	5 (1.0)	6 (1.1)	9 (1.7)	6 (1.1)	3 (0.6)	4 (0.7)
	Refused		0 (0)	0 (0)	0 (0)	2 (0.4)	0 (0)	0 (0)	0 (0)
	Total		560	505	556	520	571	531	549

^a^ Some respondents indicated that they were not interested in receiving the information; their non‐interest was noted and the remaining sub‐questions relating to their preference(s) for provision of follow‐up for that specific information topic were therefore not relevant.

For most information topics, respondents generally preferred follow‐up to be provided every 3 to 6 months, with the exception of information regarding new and/or serious side effects. About a quarter (28.2%, n = 161) preferred new and/or serious side effects to be provided as soon as possible or once the information was available.

In general, respondents were also receptive to receiving follow‐up information via email, with a higher proportion of respondents preferring this method of communication over telephone or SMS for all information topics (Table 5).

### 3.8 Broad preference for an opt‐in/opt‐out approach compared to preferences related to specific information topics

When comparing the proportions preferring an opt‐out approach when first asked and their preference in relation to a particular topic itself (Table 6), there was a significant difference in proportion of those preferring an opt‐out approach for follow‐up information about a new prescription medicine that they had recently started taking (52.4% when first asked, 59.1% when asked in relation to the specific topic, *P* = 0.001). The largest increase in proportion of those preferring an opt‐out approach was seen for information regarding new and/or serious side effects recently discovered about their medicine(s) (51.8% when first asked, 74.0% when asked in relation to the specific topic, *P* < 0.001).

**Table 6:** Comparison of broad opt‐in and opt‐out preferences in relation to preferences for specific information topics^a^

a.		Topic 1: ‘Follow‐up information about a new prescription medicine that you recently started taking’ (n = 553)				
Broad initial preference	N (%)	Opt‐in to receive this information		Opt‐out to receive this information		*P*‐value (exact sig.; 2‐sided)
		n	%	N	%	
Opt‐in	263 (47.6)	182	80.5	81	24.8	**0.001**
Opt‐out	290 (52.4)	44	19.5	246	75.2	
N (%)		226 (40.9)		327 (59.1)		
b.		Topic 2: ‘Follow‐up information about the lived experiences of others who have the same medical condition(s) as you’ (n = 498)				
Broad initial preference	N (%)	Opt‐in to receive this information		Opt‐out to receive this information		*P*‐value (exact sig.; 2‐sided)
		n	%	N	%	
Opt‐in	238 (47.8)	185	62.7	53	26.1	**<0.001**
Opt‐out	260 (52.2)	110	37.3	150	73.9	
N (%)		295 (59.2)		203 (40.8)		
c.		Topic 3: ‘Updates on new medicines or treatments that have become available on the market’ (n = 550)				
Broad initial preference	N (%)	Opt‐in to receive this information		Opt‐out to receive this information		*P*‐value (exact sig.; 2‐sided)
		n	%	N	%	
Opt‐in	265 (48.2)	173	71.5	92	29.9	0.083
Opt‐out	285 (51.8)	69	28.5	216	70.1	
N (%)		242 (44.0)		308 (56.0)		
d.		Topic 4: ‘Online websites/materials/social support groups for your medical condition(s)’ (n = 513)				
Broad initial preference	N (%)	Opt‐in to receive this information		Opt‐out to receive this information		*P*‐value (exact sig.; 2‐sided)
		n	%	N	%	
Opt‐in	248 (48.3)	189	60.4	59	29.5	**<0.001**
Opt‐out	265 (51.7)	124	39.6	141	70.5	
N (%)		313 (61.0)		200 (39.0)		
e.		Topic 5: ‘New and/or serious side effects recently discovered about the medicine(s) you are taking’ (n = 562)				
Broad initial preference	N (%)	Opt‐in to receive this information		Opt‐out to receive this information		*P*‐value (exact sig.; 2‐sided)
		n	%	N	%	
Opt‐in	271 (48.2)	105	71.9	166	39.9	**<0.001**
Opt‐out	291 (51.8)	41	28.1	250	60.1	
N (%)		146 (26.0)		416 (74.0)		
f.		Topic 6: ‘Reminders about how to use your medicine(s) properly—for example, how to use an inhaler device’ (n = 527)				
Broad initial preference	N (%)	Opt‐in to receive this information		Opt‐out to receive this information		*P*‐value (exact sig.; 2‐sided)
		n	%	N	%	
Opt‐in	251 (47.6)	172	63.2	79	31.0	0.135
Opt‐out	276 (52.4)	100	36.8	176	69.0	
N (%)		272 (51.6)		255 (48.4)		
g.		Topic 7: ‘Support and/or lifestyle advice that could help you self‐manage or improve your medical condition(s)’ (n = 544)				
Broad initial preference	N (%)	Opt‐in to receive this information		Opt‐out to receive this information		*P*‐value (exact sig.; 2‐sided)
		n	%	N	%	
Opt‐in	262 (48.2)	185	67.0	77	28.7	0.316
Opt‐out	282 (51.8)	91	33.0	191	71.3	
N (%)		276 (50.7)		268 (49.3)		

^a^ McNemar's test.

However, the proportions of respondents who preferred an opt‐out approach for topic‐specific follow‐up information provision were significantly lower in comparison to the proportion preferring an opt‐out approach initially for ‘Follow‐up information about the lived experiences of others who have the same medical condition(s) as you’ (52.2% when first asked, 40.8% when asked in relation to the specific topic, *P* < 0.001); and ‘Online websites/materials/social support groups for your medical condition(s)’ (51.7% when first asked, 39.0% when asked in relation to the specific topic, *P* < 0.001).

## 4 DISCUSSION

The findings of this study suggest that there is some interest among the Australian public in receiving information from their HCPs about their medicines and medical conditions outside of consultation times. Opinions, however, were divided regarding an opt‐out or opt‐in approach. Factors that may have influenced preference for an opt‐in or opt‐out approach appeared to be specific to the type of information being received.

Despite only a small proportion having received follow‐up information previously, most respondents expressed interest in receiving ongoing follow‐up information tailored to their needs. Notably, respondents who had received previous follow‐up information were more likely to be interested in receiving follow‐up information outside of consultations in the future. Those who self‐reported needing help reading information, and difficulty learning about their medical conditions and/or medicines due to reading difficulties, were more interested in receiving follow‐up information. These findings highlight the need for information, expressed by patients, which has been noted in past research,^29^, ^30^, ^31^, ^32^ and the fact that HCPs are not meeting the needs of patients by providing tailored information targeted to their perceived needs.^29^, ^33^, ^34^ Importantly, the results show that the groups who would be more interested could be targeted for opt‐out information provision.

General practice standards in Australia have addressed the engagement of telephone and/or electronic communications under appropriate circumstances and within clearly defined parameters between the practice and their patients.^35^ Thus, although an opt‐out approach to provision of follow‐up is not currently embedded within the Australian health‐care system, there is opportunity for GP practices to proactively engage in the provision of tailored information outside of consultation times to help improve existing information exchanges. This also extends to other international contexts where opt‐in approaches to further information provision are presently implemented.

On the other hand, respondents not interested in receiving follow‐up tailored information believed that they already received the information they needed during consultations, and/or that they would not benefit from further information since they were adherent to their medicines. This is consistent with previous studies relating to health and/or medicines information seeking.^36^, ^37^ Similarly, preference for follow‐up information provision by their GP by a majority of those who were interested in receiving follow‐up is also consistent with the literature regarding patients’ key sources of health and/or medicines information.^37^, ^38^

Respondents in the present study were almost equally divided in their preference for the receipt of ongoing follow‐up information outside of consultation times and provided similar rationale for their choice, as observed in previous focus group discussions.^18^ As would be expected, those participants who were interested in receiving follow‐up information were more likely to prefer an opt‐out process, since this would ensure that they received the desired follow‐up information.

Preferences for an opt‐in versus opt‐out service differed in relation to specific topics of information. A higher proportion of respondents preferred an opt‐out approach for receiving follow‐up information about a new medicine, including updates, and information about new and/or serious side effects compared with those who preferred an opt‐out approach in general. A possible explanation is that some respondents, while opposing the principle of opt‐out in general, consider that specific topics are more salient to them and have a bearing on medication safety and quality of life (eg. side effects).^2^, ^39^ From a systems perspective, an opt‐out approach would ensure that all patients would receive this information. When communicated in a tailored, unbiased and non‐alarming way, such information could support patients to become better informed about their own health. The potential for increased reach and directed communication of health‐related information to the public may then in turn contribute towards benefits at an organizational and system level with respect to the quality and timeliness of information provision. Furthermore, an opt‐out system would allow for timely dissemination of information that is relevant to medication safety, for example pharmacovigilance updates, and may also have positive flow‐on effects to other post‐marketing surveillance efforts, for example increased consumer reporting of adverse drug events. In contrast, preference for an opt‐in service for follow‐up information about other people's lived experiences, and online websites and social support groups, indicates that this information was regarded as less important. This suggests that a blanket opt‐out approach to health‐related information provision outside of consultation times may be unnecessary. The relative topic‐specific importance from the patient perspective, highlighted in the present study, may help to inform a more targeted approach for legislative change that can then support changes in information provision practice.

Interestingly, there was no consensus about the frequency of providing consent as part of an opt‐in process. In contrast, in earlier focus groups, among participants who preferred an opt‐in approach, there was general agreement that consent would only need to be provided once for a HCP to provide information on an ongoing basis.^18^ This difference may indicate a reluctance to apply a blanket approach regarding consent among the broader population. It may also represent a more conservative approach whereby providing consent at regular intervals will better meet changing information needs throughout the treatment continuum. Additionally, in the focus groups, it was possible to obtain consensus through discussion and exchange of ideas resulting in a reasonable decision to be reached by the group. In the present study, however, individuals gave their opinions without further discussion.

Although current Australian legislation requires consent to be given by patients to receive follow‐up information from HCPs (ie an opt‐in system), the survey results suggest that a move to an opt‐out process may be well received by the Australian public. In a patient‐centered health‐care system, patients have a right to receive information to assist them in optimal self‐management and care. Moreover, patients should take responsibility and exercise autonomy when deciding whether they want or need the information being offered, rather than rely on HCPs alone to make that decision for them. An opt‐out process may encourage more informed and shared decision making in health, in partnership with patients and their HCPs. Although GPs were consistently nominated as preferred follow‐up information providers, previous studies involving cancer patients/survivors found that the specialist was the preferred HCP to provide follow‐up.^17^, ^40^, ^41^, ^42^ Therefore, preferences for providers of information may also be condition dependent. This, together with receptivity for an opt‐out approach to follow‐up information provision, should be further evaluated as part of future research.

This study had several limitations. The overall original sample stratification target quotas were met, apart from the age quotas where a lower proportion of persons aged 18‐34 years participated in the survey. As the survey did not screen for the level of health literacy, and therefore did not actively recruit people across a range of health literacy levels, the study findings will not be generalizable to certain subpopulations such as people with low to poor health literacy. This survey did not intend to collect data on the participants’ specific medical conditions. Thus, it is not possible to determine the influence of medical conditions on the level of interest and preference for an opt‐in versus opt‐out approach.

## 5 CONCLUSIONS

Members of the Australian public are interested in receiving information outside of consultation times, with this information being tailored to their needs. However, preferences for an opt‐in or opt‐out approach were clearly divided.

Previous lack of receiving follow‐up information coupled with interest in receiving follow‐up information from their HCPs indicates that there are unmet needs among patients for further information. However, further exploration as to whether there should be a transition towards a blanket opt‐out approach is required prior to implementation. This exploration should involve all key stakeholders, such as HCPs and policy makers, and should also consider resource needs.

## CONFLICT OF INTEREST

At the time of the study, Stephen Robson was employed by the Healthy Thinking Group P/L. Vivien Tong, Ines Krass, and Parisa Aslani have no conflicts of interest to declare.

## AUTHOR CONTRIBUTIONS

Vivien Tong: Research Design and Methods; Data analysis; Writing – Original draft; Writing – review and editing; Project Administration. Ines Krass: Conceptualization, Research Design and Methods; Data analysis; Writing – review and editing; Funding Acquisition. Stephen Robson: Conceptualization, Writing – review and editing. Parisa Aslani: Conceptualization, Research Design and Methods; Data analysis; Writing – review and editing; Funding Acquisition; Project Administration.

## Data Availability

The study data are not available as the respondents to the survey have not provided consent for data to be made available beyond the research team. Furthermore, the research team only has the respondents’ consent to publish de‐identified group data.
